# Proteomic characterization of iron deficiency responses in *Cucumis sativus *L. roots

**DOI:** 10.1186/1471-2229-10-268

**Published:** 2010-12-01

**Authors:** Silvia Donnini, Bhakti Prinsi, Alfredo S Negri, Gianpiero Vigani, Luca Espen, Graziano Zocchi

**Affiliations:** 1 Dipartimento di Produzione Vegetale, Università degli Studi di Milano, Via Celoria 2, 20133 Milano, Italy

## Abstract

**Background:**

Iron deficiency induces in Strategy I plants physiological, biochemical and molecular modifications capable to increase iron uptake from the rhizosphere. This effort needs a reorganization of metabolic pathways to efficiently sustain activities linked to the acquisition of iron; in fact, carbohydrates and the energetic metabolism has been shown to be involved in these responses. The aim of this work was to find both a confirmation of the already expected change in the enzyme concentrations induced in cucumber root tissue in response to iron deficiency as well as to find new insights on the involvement of other pathways.

**Results:**

The proteome pattern of soluble cytosolic proteins extracted from roots was obtained by 2-DE. Of about two thousand spots found, only those showing at least a two-fold increase or decrease in the concentration were considered for subsequent identification by mass spectrometry. Fifty-seven proteins showed significant changes, and 44 of them were identified. Twenty-one of them were increased in quantity, whereas 23 were decreased in quantity. Most of the increased proteins belong to glycolysis and nitrogen metabolism in agreement with the biochemical evidence. On the other hand, the proteins being decreased belong to the metabolism of sucrose and complex structural carbohydrates and to structural proteins.

**Conclusions:**

The new available techniques allow to cast new light on the mechanisms involved in the changes occurring in plants under iron deficiency. The data obtained from this proteomic study confirm the metabolic changes occurring in cucumber as a response to Fe deficiency. Two main conclusions may be drawn. The first one is the confirmation of the increase in the glycolytic flux and in the anaerobic metabolism to sustain the energetic effort the Fe-deficient plants must undertake. The second conclusion is, on one hand, the decrease in the amount of enzymes linked to the biosynthesis of complex carbohydrates of the cell wall, and, on the other hand, the increase in enzymes linked to the turnover of proteins.

## Background

Iron is an essential element for all living organisms, being part of many proteins participating in fundamental mechanisms such as DNA synthesis, respiration, photosynthesis and metabolism [1]. In plants, the main cause of Fe deficiency is its low availability in the soil solution due to the scarce solubility of its compounds in well aerated environments. To cope with this problem plants have developed efficient mechanisms to acquire Fe from the soil. Two main strategies are known: dicots and non-graminaceous monocots operate applying what is known as Strategy I, while graminaceous monocots operate with the so-called Strategy II [2,3]. In the last decade a great amount of biochemical and molecular data have been acquired, increasing the knowledge about the mechanisms adopted by Strategy I plants, especially when grown in the absence of Fe. In particular, three main events seem to assure iron uptake. First, the induction of the reducing activity of a Fe^3+^-chelate reductase (FC-R) located at the plasma membrane of epidermal root cells. The enzyme was first cloned in *Arabidopsis *(*AtFRO2*) [4] and *FRO2 *homologues were found in other Strategy I plants [5-7]; second, the induction of a Fe^2+ ^transporter belonging to the ZIP family of proteins [8] and identified as IRTs in several plants [9,10]; third, the activation of a P-type H^+^-ATPase [11-13] necessary to decrease the apoplastic pH, thus favouring, on one hand, the solubilization of external Fe compounds and the activity of the FC-R [14,15] and, on the other hand, to establish an effective driving force for Fe uptake [11,16,17]. Since the maintenance of these activities requires the constant production of energetic substrates, changes in metabolism have also been studied under Fe deficiency conditions. It has been shown that the rate of glycolysis is increased [18,19]; the pentose phosphate pathway is increased, as well, to produce both reducing equivalents and carbon skeletons [18,20]. Furthermore, the phospho*enol*pyruvate carboxylase (PEPC) activity has been shown to increase several times under Fe deficiency [21,22]. This enzyme is very important in the economy of the cell, since it can accomplish several tasks: (i), by consuming PEP it increases the rate of glycolysis, releasing the negative allosteric control exerted on phosphofructo kinase-1 (PFK-1) and aldolase by this phosphorylated compound [23]; (ii), it contributes to the intracellular pH-stat mechanisms [24] and (iii), it forms organic acids, in particular malate and citrate, that may play an important role in the transport of iron through the xylem to the leaf mesophyll [25,26]. Furthermore, PEPC activity sustains the anaplerotic production of carbon skeletons for biosynthetic pathways (in particular the synthesis of amino acids) and along with the accumulation of di-tricarboxylic acid carrier (DTC), increases the communication between the cytosolic and mitochondrial pools of organic acids, to help maintaining a higher turnover of reducing equivalents [27]. Implication of metabolism has been also inferred from the microarray analysis performed on Fe-starved *Arabidopsis *plants [28], in which it was shown that the levels of several transcripts encoding enzymes of these metabolic pathways were increased. However, the changes in transcript levels are not a direct proof that the encoded proteins have changed, but that relevant metabolic pathway or biological processes have been affected. To study a global change in the concentration of proteins the new proteomic technologies can be undoubtedly of great help. Concerning plant iron nutrition, two recent studies have analysed by 2-DE the proteome of wild-type tomato and its *fer *mutant [29,30] grown under Fe deficiency, to identify to what extent the transcription factor FER influences the accumulation of Fe-regulated protein, while another one analysed the changes in proteomic and metabolic profiles occurring in sugar beet root tips in response to Fe deficiency and resupply [31].

Cucumber (*Cucumis sativus *L.) plants develop rapid responses to Fe deficiency, and previous works by our and other groups have described very important changes, not only in the classical responses of Strategy I plants, i.e. FC-R and H^+^-ATPase activities, but also in the metabolic rearrangement induced by Fe starvation [7,18,19,32,33].

In this work we have carried out a proteomic analysis on proteins isolated from cucumber roots grown in the presence or in the absence of Fe for 5 and 8 d. Furthermore, we chose to analyse only the cytosolic soluble protein fraction without contaminations by organelles or membranes.

## Results

### Experimental planning and 2-DE analysis

**Figure 1:**
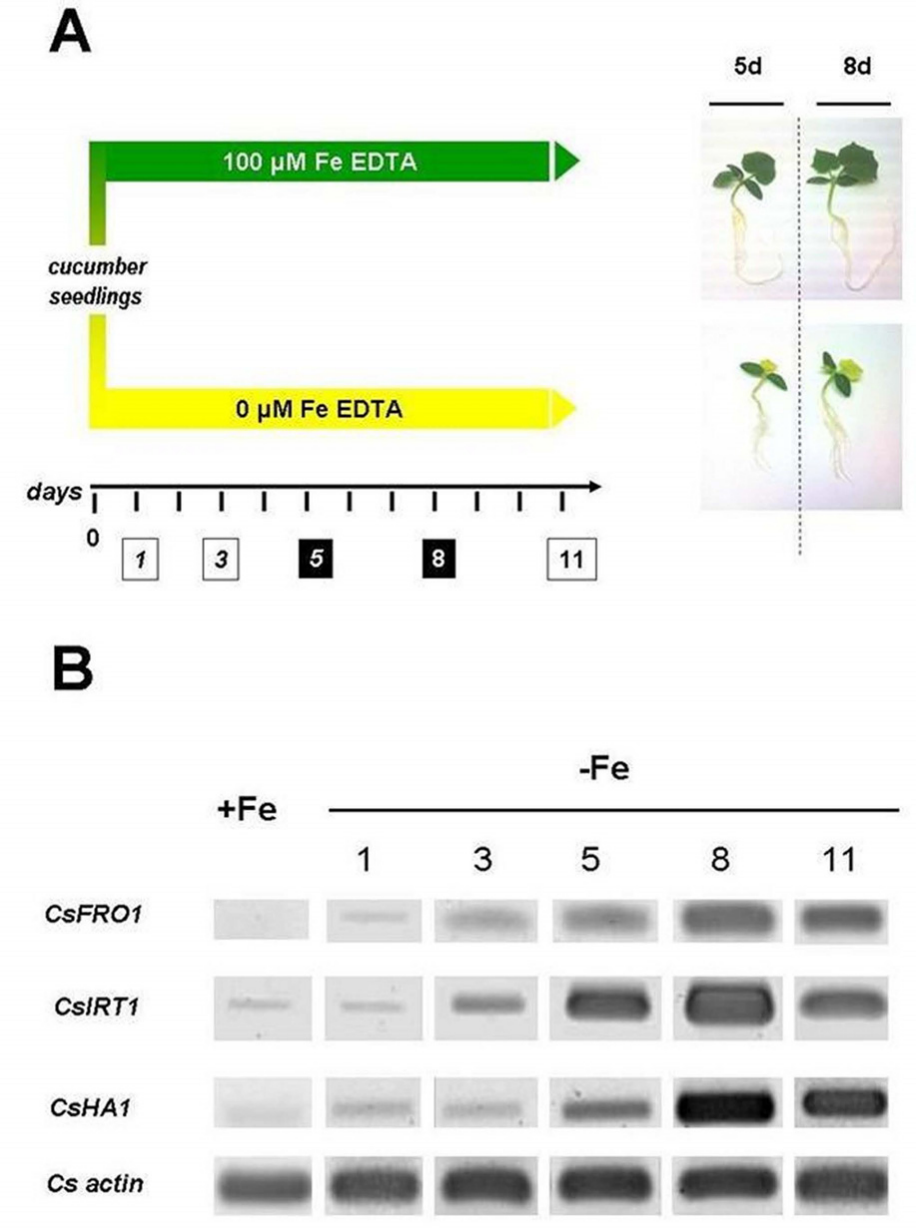
**Experimental plan and RT-PCR analysis**. (A) Scheme of the growth conditions used in this work: white rectangles (1, 3, 11) indicate the time, after the induction of Fe deficiency, at which plant root apexes were sampled only for RT-PCR semiquantitative analysis reported in (B); black rectangles (5 and 8) indicate the time at which the root apexes of Fe-deficient (-Fe) plants were sampled only for RT-PCR semiquantitative analysis reported in (B) and whole roots for the proteomic analysis. On the right, pictures of plants under the different growing conditions are shown. (B) semi-quantitative RT-PCR analysis of the genes *CsFRO1 *(encoding for FC-R), *CsIRT1 *(encoding for the IRT1) and *CsHA1 *(encoding for the H^+^-ATPase) in cucumber root under the different treatments. The column +Fe represents results for control plants grown in the presence of iron. The columns -Fe 1, 3, 5, 8, 11 represent results for days after -Fe treatment induction at which the roots were sampled as specified in the panel A.

**Figure 2:**
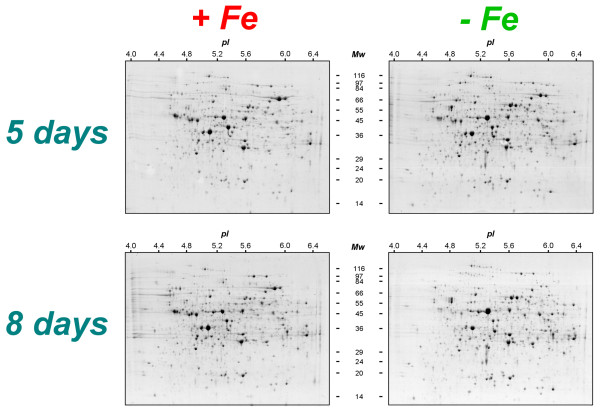
**2-DE maps**. 2-DE maps of soluble protein fractions extracted from roots of cucumber plants grown for 5 and 8 d in the presence (+Fe) or absence (-Fe) of Fe. Proteins (400 μg per gel) were separated by IEF at pH 4-7, followed by 10% SDS PAGE and visualized by cCBB-staining. The number of spots detected was 2029 ± 272 for -Fe 5 d, 2136 ± 330 for +Fe 5 d, 1999 ± 223 for -Fe 5 d and 2208 ± 168 for +Fe 8 d.

In this study the changes in the protein profile of cucumber roots expressed in response to Fe deficiency were analyzed. The choice to collect proteins after 5 and 8 days of growth was done after a preliminary analysis in which we assessed the increases in transcript abundances related to the Strategy I adaptation responses occurring under Fe-starvation (Figure 1A and 1B) and by previous biochemical evidence obtained by our laboratory [18,19,34]. Figure 1B shows the rapid increase occurring for the mRNAs encoding for the three typical Strategy I proteins. While for *CsFRO1 *and *CsIRT1 *their expression increased strongly at early stages, for *CsHA1 *the increase occurred later after Fe deficiency induction. Eight-d-old plants showed the highest response for all three transcripts. Soluble (cytosolic) proteins were obtained from roots of plants grown in the presence or in the absence of Fe, after centrifugation to eliminate any possible contamination by organelles and endomembranes. Proteins were successively separated by 2-DE. Figure 2 reports the two-dimensional gel electrophoresis representative maps of soluble proteins isolated from roots of plants grown for 5 and 8 d in the presence or in the absence of Fe.

### Hierarchical clustering analysis

**Figure 3:**
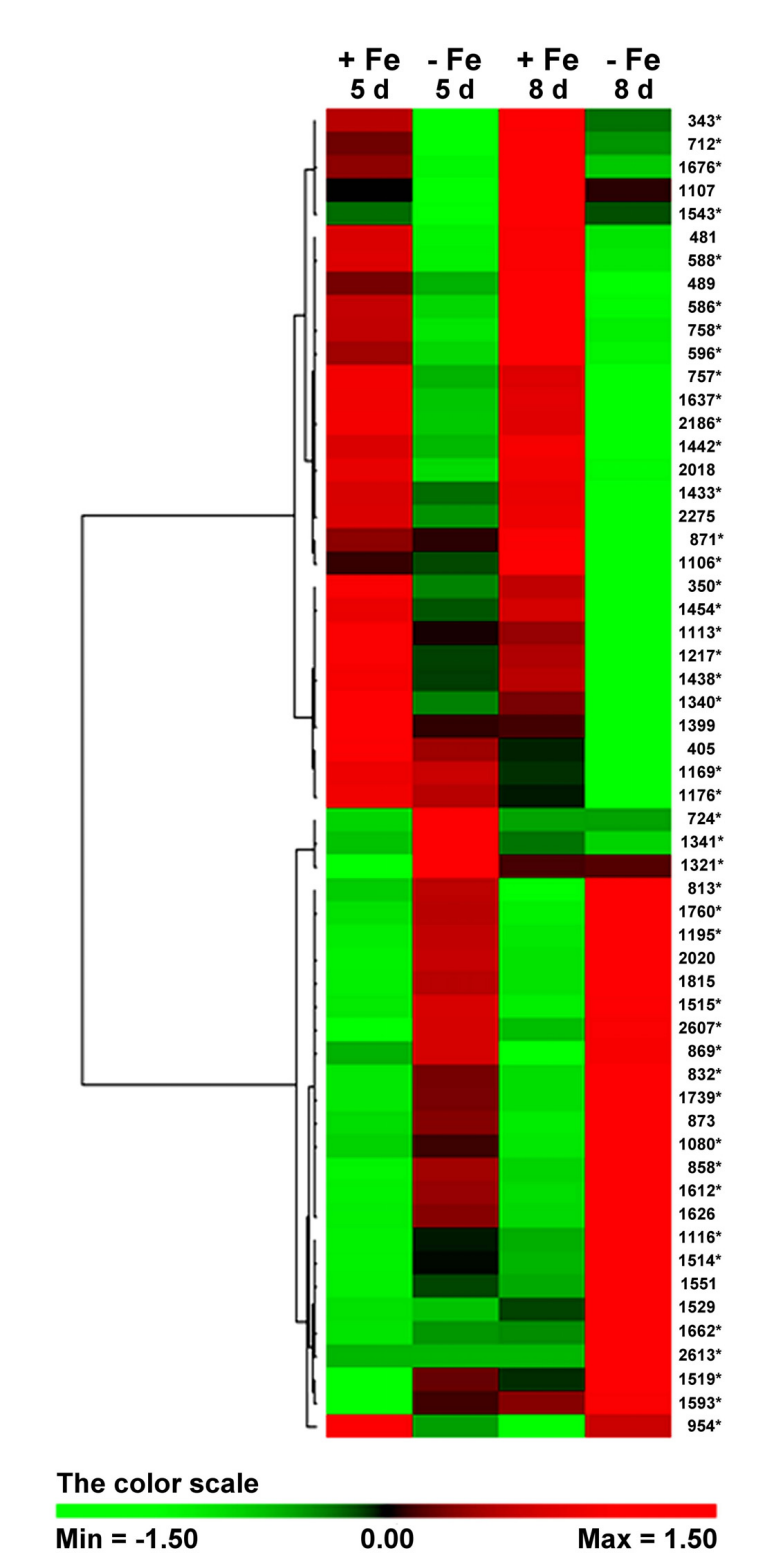
**Clustering analysis**. Two-way hierarchical clustering analysis of the 57 spots that showed at least a two-fold change in the relative spot volumes (Two-ways ANOVA, p > 0.001) with Fe and days of treatment as factors. The clustering analysis was performed with PermutMatrix graphical interface after Z-score normalization of the averages of relative spot values (n = 6). Pearson's distance and Ward's algorithm were used for the analysis. Each coloured cell represents the average of the relative spot value, according to the colour scale at the bottom of the figure. Spots labelled with asterisks are those subsequently identified by MS/MS.

The comparison between the control and the -Fe treatment showed that 57 protein spots were expressed differentially. These spots were subjected to two-way hierarchical clustering analysis using the PermutMatrix software [35]. Figure 3 represents the results obtained and shows the pairwise comparison of protein levels for the two dates and the two Fe treatments chosen. The protein spots were sorted in two main groups: those showing a decreased abundance in the presence of Fe and those which accumulate in the presence of the ion. Focusing the attention on lower level groupings, it is interesting to note that the protein behavior at the two dates was quite similar but not identical, because although most differences were more marked after 8 d, some other ones (e.g. spots 724, 1341, 1321) were essentially associated to the 5-d stage. These evidences underlined that cucumber root response can be slightly but significantly affected by some peculiar traits depending on the considered stage of Fe deficiency.

### Comparative analysis of the soluble proteins under Fe deficiency

**Figure 4:**
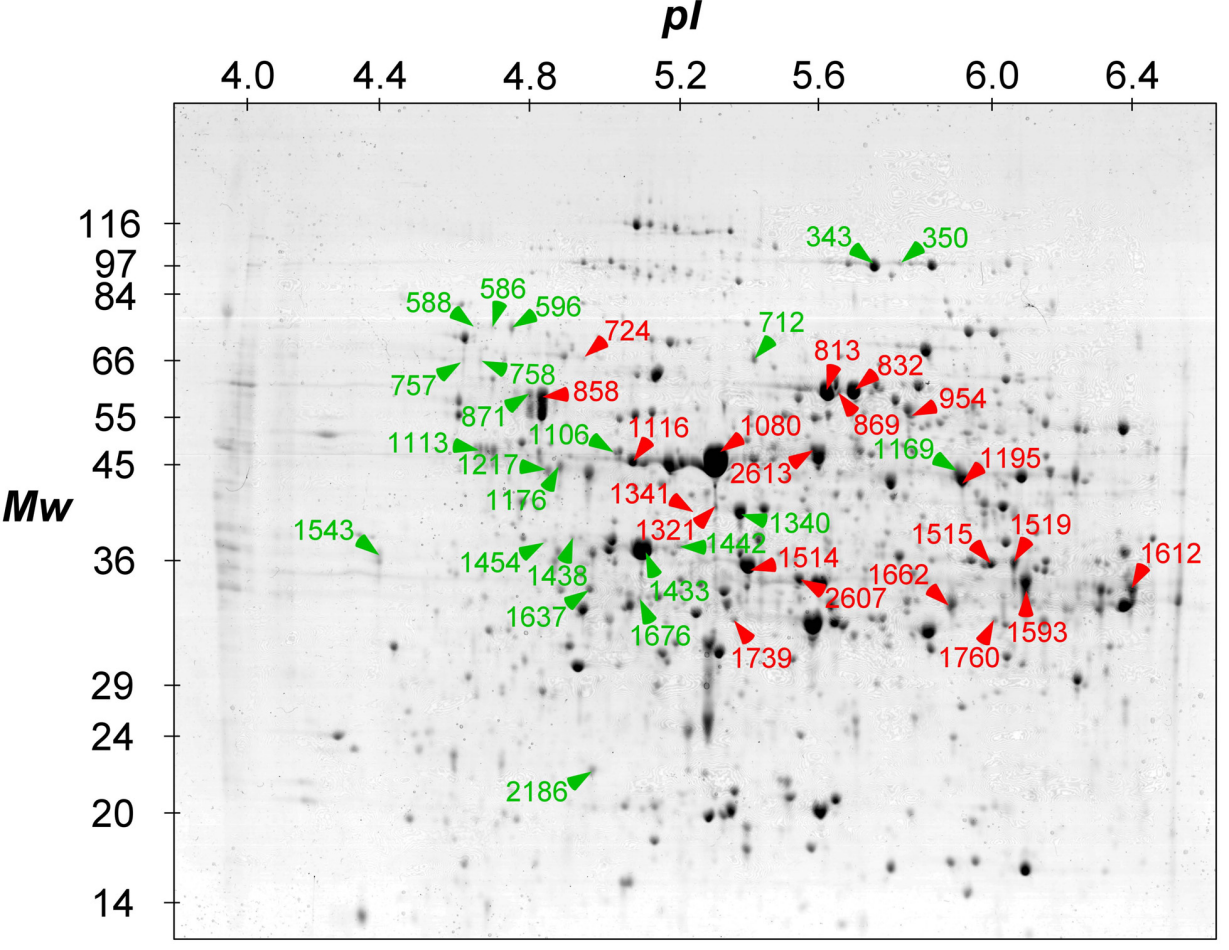
**2-DE map of identified proteins**. Representative 2-DE map of the proteins of interest in the soluble fraction extracted from cucumber roots obtained from plants grown for 5, and 8 d in the presence (+Fe) or absence (-Fe) of Fe. Proteins were analyzed by IEF at pH 4-7, followed by 10% SDS PAGE and visualized by cCBB-staining. Numbers corresponding to those in Table 1 and Table 2, indicate the spots identified by LC-ESI-MS/MS. Proteins that increased or decreased under Fe deficiency are reported in red and in green, respectively.

The 57 spots of interest were analyzed by LC-ESI-MS/MS. Forty-four out of them were identified and listed in Tables 1 and 2 and indicated by numbers in Figure 4. Numbers in red in Figure 4 identified proteins whose amount is increased, while the numbers in green identified proteins whose amount is decreased under Fe deficiency. Statistical information about LC-ESI-MS/MS analysis are reported in Additional file 1.

**Table 1:** List of the 21 proteins identified by LC-ESI-MS/MS whose concentration is increased under Fe deficiency in cucumber roots.

Spot ID	Accession number	Species	Protein description	EC	Abbreviation	**M**_**r **_^***a***^**/pI **^***a***^	**M**_**r **_^***b***^**/pI **^***b***^	**Cov. (%) **^***c***^
**Glycolysis**								
**813**	Q42908	*Mesembryanthemum crystallinum*	2,3-bisphosphoglycerate-independent phosphoglycerate mutase	5.4.2.1	PGAM1-a	60.0/5.6	61.2/5.4	18
**832**	O24246	*Prunus dulcis*	2,3-bisphosphoglycerate-independent phosphoglycerate mutase	5.4.2.1	PGAM1-b	60.0/5.6	53.4/5.4^*d*^	20^*d*^
**869**	P35493	*Ricinus communis*	2,3-bisphosphoglycerate-independent phosphoglycerate mutase	5.4.2.1	PGAM1-c	60.0/5.6	60.8/5.5	10
**954**	Q41141	*Ricinus communis*	pyrophosphate--fructose 6-phosphate 1-phosphotransferase subunit beta	2.7.1.90	PPi-PFK	54.4/5.8	60.1/6.2	5
**1080**	P42896	*Ricinus communis*	Enolase	4.2.1.11	ENO-a	44.9/5.3	47.9/5.6	42
**1116**	AAS66001	*Capsella bursa-pastoris*	LOS2	4.2.1.11	ENO-b	46.4/5.1	47.7/5.4	32
**1514**	Q42962	*Nicotiana tabacum*	phosphoglycerate kinase, cytosolic	2.7.2.3	PGK	36.7/5.4	42.4/5.7	44
**1612**	CAB77243	*Persea americana*	fructose-bisphosphate aldolase	4.1.2.13	FBA-a	35.4/6.4	38.6/6.5	20
**1662**	CAB77243	*Persea americana*	fructose-bisphosphate aldolase	4.1.2.13	FBA-b	34.6/5.9	38.6/6.5	20
**Carbohydrate-related metabolism**								
**1519**	ABC02081	*Cucumis melo*	putative alcohol dehydrogenases	1.1.1.1	ADH-a	36.9/6.0	41.0/6.8	26
**1593**	ABC02081	*Cucumis melo*	putative alcohol dehydrogenases	1.1.1.1	ADH-b	35.7/6.1	41.0/6.8	20
**1739**	Q08062	*Zea mays*	malate dehydrogenase, cytoplasmic	1.1.1.37	MDH	33.7/5.3	35.6/5.8	7
**2613**	ACJ04703	*Cucumis melo*	galactokinase	2.7.1.6	GALK	49.2/5.6	54.6/5.7	20
**Nitrogen-related metabolism**								
**1195**	AAR05449	*Capsicum annuum*	alanine aminotransferase	2.6.1.2	AAT	43.3/5.9	52.8/5.3	10
**1321**	A9P822	*Populus trichocarpa*	S-adenosylmethionine synthetase 1	2.5.1.6	MAT1-a	40.7/5.3	43.2/5.7	17
**1341**	AAT40304	*Medicago sativa*	S-adenosylmethionine synthase	2.5.1.6	SAMs	40.6/5.3	42.8/5.7	28
**1760**	NP_196765	*Arabidopsis thaliana*	carbon-nitrogen hydrolase family protein	3.5.-.-	CNH	33.3/6.0	40.3/8.8	14
**2607**	P51118	*Vitis vinifera*	glutamine synthetase cytosolic isozyme 1	6.3.1.2	GS1	36.0/5.5	39.2/5.8	29
**Redox-related and other proteins**								
**724**	CAB72130	*Cucumis sativus*	heat shock protein 70	- - -	HSP70-a	67.1/4.9	70.8/5.3	30
**858**	AAU04766	*Cucumis melo*	protein disulfide isomerase (PDI)-like protein 2	5.3.4.1	PDI2-a	58.1/4.8	63.7/5.0	10
**1515**	CAN60665	*Vitis vinifera*	old yellow enzyme-like^*e*^	1.6.99.1	OYE	37.0/6.0	42.0/5.8	8

Proteins were classified on the basis of data available in the literature. Statistical information about LC-ESI-MS/MS analysis are reported in Additional file 1.

^***a***^: experimental molecular weight (kDa) or isoelectric point.

^***b***^: theoretical molecular weight (kDa) or isoelectric point.

^***c***^: amino acid coverage (%).

^*d*^: partial sequence.

^*e*^: annotation reported by the authors.

Some of the proteins were identified in more than one spot in the 2-DE gel. The variability in the level of proteins belonging to the same family suggests the presence of different isoforms, which can be subjected to different post-translational modifications.

**Figure 5:**
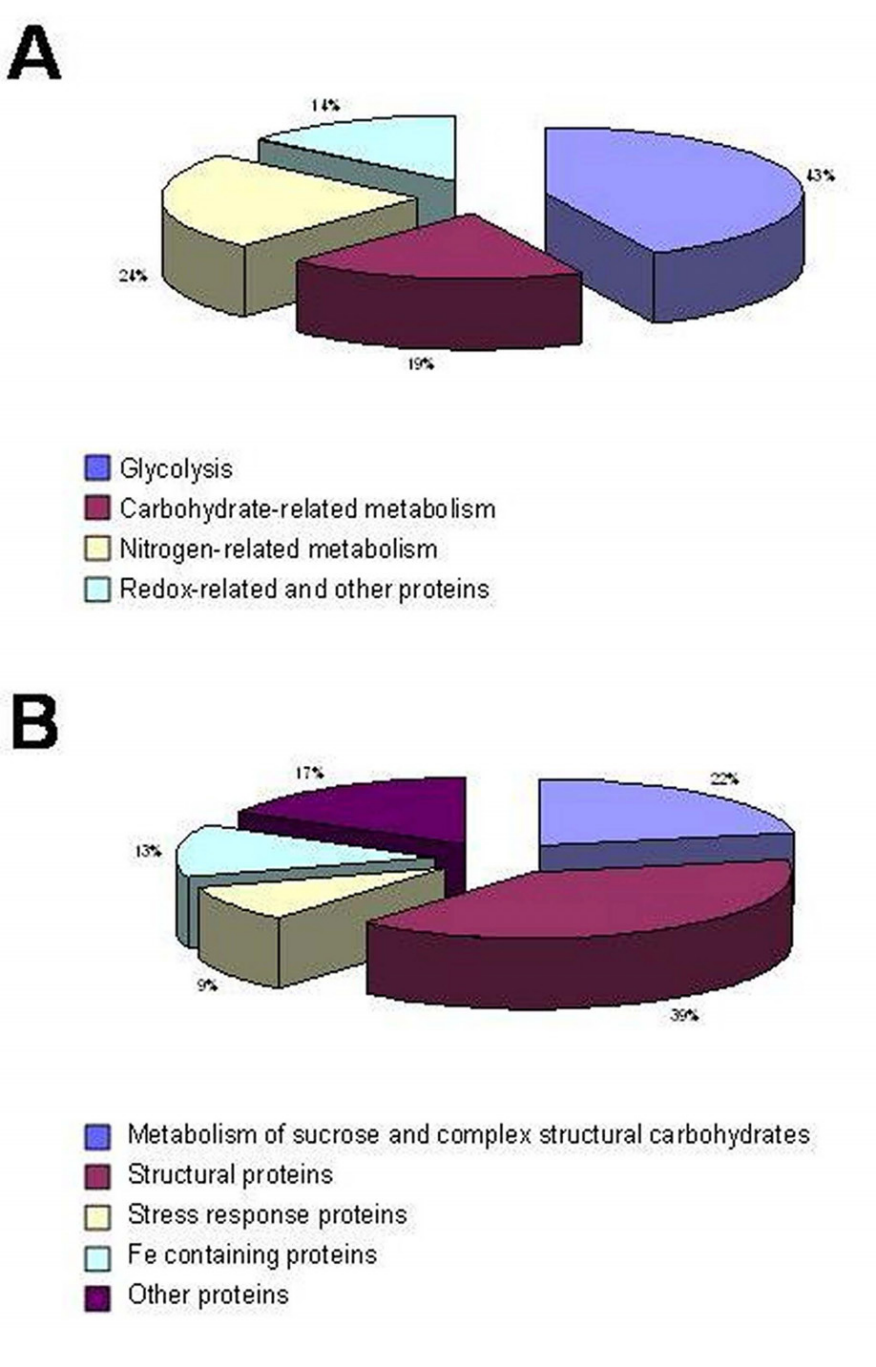
**Functional categories distribution of the identified proteins**. Functional distribution of identified proteins according to the data available in the literature. A. proteins whose concentration is increased under Fe deficiency; B. proteins whose concentration is decreased under Fe deficiency.

Twenty-one protein spots out of 44 showed increased accumulation (Table 1) in the absence of Fe with a further increase between the pairwise comparison after 8 d (Figure 3). The increased proteins under Fe deficiency were sorted into four different functional classes (Figure 5A) on the basis of data available in the literature. All the identified proteins except one (spot number 724) were characterized as enzymes and most of them (43%) belong to the glycolytic/gluconeogenetic pathways, confirming the proteomic [29-31] and the biochemical data obtained by several groups [18,19,22] and the prediction from microarray analysis of Fe-deficient *Arabidopsis *[28]. We have also considered that the spot number 954 (the pyrophosphate-fructose-6-phosphate 1-phosphotransferase) belongs to this group, since under Fe deficiency it follows the increasing trend shown by other glycolytic enzymes. In fact, after 8 d there is a substantial increase in the level of this protein notwithstanding an initial decrease. This increase is corroborate by the enzymatic assay that show that after 8 d of Fe deficiency the activity is increased two-fold (data not shown). A second group of proteins (19% of the total) were classified as belonging to the general carbohydrate metabolism. In this group we have included the spot identified as malate dehydrogenase (number 1739) and two spots corresponding to alcohol dehydrogenase (number 1519 and 1593). Among them, one spot (number 2613) is of particular interest since it appears only after 8 d of Fe deficiency and was identified as a galactokinase. A third group (24%) belongs to nitrogen metabolism and includes alanine aminotransferase (spot number 1195), two spots corresponding to S-adenosyl methionine synthase (number 1321 and 1341), glutamine synthase 1 (number 2607) and a spot identified as a C-N hydrolase (number 1760). The last 14% of the proteins belongs to cellular redox proteins and other. One spot (number 724) corresponds to a heat shock protein 70, while the other two spots match with a disulfide isomerase protein (PDI, number 858), which catalyses the formation, isomerization and reduction/oxidation of disulfide bonds [36] and with an old yellow enzyme-like protein (OYE) (number 1515) that was the first enzyme shown to contain flavins as cofactor. Proteins from OYE family can use either NADPH, NADH or both, thus classifying them as NAD(P)H oxidoreductase [37].

Twenty-three out of 44 protein spots identified were decreased in quantity (Table 2) under Fe deficiency. Among these 11 were characterized as enzymes and 13 as structural or stress response proteins. The proteins decreased in quantity were also sorted into five different functional classes according to the literature (Table 2 and Figure 5B), with some proteins (22%) involved in the metabolism of sucrose and complex structural carbohydrates, such as invertase (spots number 586, 588, 596), 1,4-β-xylosidase (spot 712) and UDP-glucose dehydrogenase (spot 1169). A second group (39%) has been identified as structural proteins (spots number 1113, 1176, 1217, 1433, 1438, 1442, 1454, 1637 and 1676) and a third one (9%) as stress-response proteins (spots number 757 and 758). The fourth group (13%) comprises proteins containing Fe, such as aconitase (number 349 and 350) and peroxidase (number 1543). The last group (17%) contains a PDI-like protein (spot 871), the beta subunit of the mitochondrial ATPase (spot 1106), a S-adenosylmethionine synthase (spot 1340) and a wali7-like protein (spot 2186).

**Table 2:** List of the 23 proteins identified by LC-ESI-MS/MS whose concentration is decreased under Fe deficiency in cucumber roots.

Spot ID	Accession number	Species	Protein description	EC	Abbreviation	**M**_**r **_^***a***^**/pI**^***a***^	**M**_**r **_^***b***^**/pI**^***b***^	**Cov. (%)**^***c***^
**Metabolism of sucrose and complex structural carbohydrates**								
**586**	ACJ04702	*Cucumis melo*	invertase 2	3.2.1.26	INV2-a	72.6/4.7	69.7/4.9	11
**588**	ACJ04702	*Cucumis melo*	invertase 2	3.2.1.26	INV2-b	73.1/4.7	69.7/4.9	7
**596**	ACJ04702	*Cucumis melo*	invertase 2	3.2.1.26	INV2-c	72.5/4.7	69.7/4.9	11
**712**	CAJ65921	*Populus alba *x *Populus tremula*	xylan 1,4-beta-xylosidase	3.2.1.37	β-Xilosidase	67.8/5.4	75.8/5.2	5
**1169**	CAN62897	*Vitis vinifera*	predicted UDP-glucose 6-dehydrogenase^*d*^	1.1.1.22	UDPGDH	44.2/5.9	53.0/6.4	15
**Structural proteins**								
**1113**	ABS50668	*Eucalyptus grandis*	beta-tubulin	- - -	β-TUB	45.7/4.7	50.5/4.7	26
**1176**	P22275	*Zea mays*	tubulin alpha-3 chain	- - -	α-TUB-a	43.3/4.9	49.6/5.1	30
**1217**	AAO73546	*Ceratopteris richardii*	alpha-tubulin	- - -	α-TUB-b	42.9/4.8	49.7/4.9	20
**1433**	AAP73449	*Gossypium hirsutum*	actin	- - -	ACT-a	37.4/5.1	41.7/5.3	47
**1438**	AAG10041	*Setaria italica*	actin	- - -	ACT-b	38.2/4.9	41.7/5.3	29
**1442**	AAP73449	*Gossypium hirsutum*	actin	- - -	ACT-c	38.1/5.2	41.7/5.3	27
**1454**	AAP73449	*Gossypium hirsutum*	actin	- - -	ACT-d	38.0/4.8	41.7/5.3	18
**1637**	AAF64423	*Cucumis melo*	globulin-like protein	- - -	Globulin	34.9/4.7	19.9/4.9^*e*^	7^*e*^
**1676**	AAP73449	*Gossypium hirsutum*	actin	- - -	ACT-e	34.6/5.1	41.7/5.3	18
**Stress response proteins**								
**757**	CAB72130	*Cucumis sativus*	heat shock protein 70	- - -	HSP70-b	66.0/4.6	70.8/5.3	24
**758**	CAB72129	*Cucumis sativus*	heat shock protein 70	- - -	HSP70-c	66.2/4.7	71.5/5.1	16
**Fe containing proteins**								
**343**	P49608	*Cucurbita maxima*	aconitate hydratase, cytoplasmic	4.2.1.3	ACO-a	96.0/5.7	98.0/5.7	9
**350**	AAC26045	*Citrus limon*	aconitase-iron regulated protein 1	4.2.1.3	ACO-b	5.8/97.5	98.1/5.9	8
**1543**	AAA33129	*Cucumis sativus*	peroxidase	1.11.1.7	POX	36.6/4.4	31.9/4.7^*f*^	17^*f*^
**Other proteins**								
**871**	AAU04766	*Cucumis melo*	protein disulfide isomerase (PDI)-like protein 2	5.3.4.1	PDI2-b	59.1/4.8	63.7/5.0	9
**1106**	P19023	*Zea mays*	ATP synthase subunit beta, mitochondrial	3.6.3.14	ATP-β	47.3/5.0	54.1/5.2^*f*^	22^*f*^
**1340**	A9P822	*Populus trichocarpa*	S-adenosylmethionine synthetase 1	2.5.1.6	MAT1-b	40.1/5.3	43.2/5.7	31
**2186**	CAN71784	*Vitis vinifera*	wali7-like protein^*d*^	- - -	W7	22.2/5.0	27.2/5.6	9

Proteins were classified on the basis of data available in the literature. Statistical information about LC-ESI-MS/MS analysis are reported in Additional file 1.

^***a***^: experimental molecular weight (kDa) or isoelectric point.

^***b***^: theoretical molecular weight (kDa) or isoelectric point.

^***c***^: amino acid coverage (%).

^*d*^: annotation reported by the authors.

^*e*^: partial sequence.

^*f*^: values referred to the mature form of the protein.

### Change in the protein level under Fe deficiency

**Figure 6:**
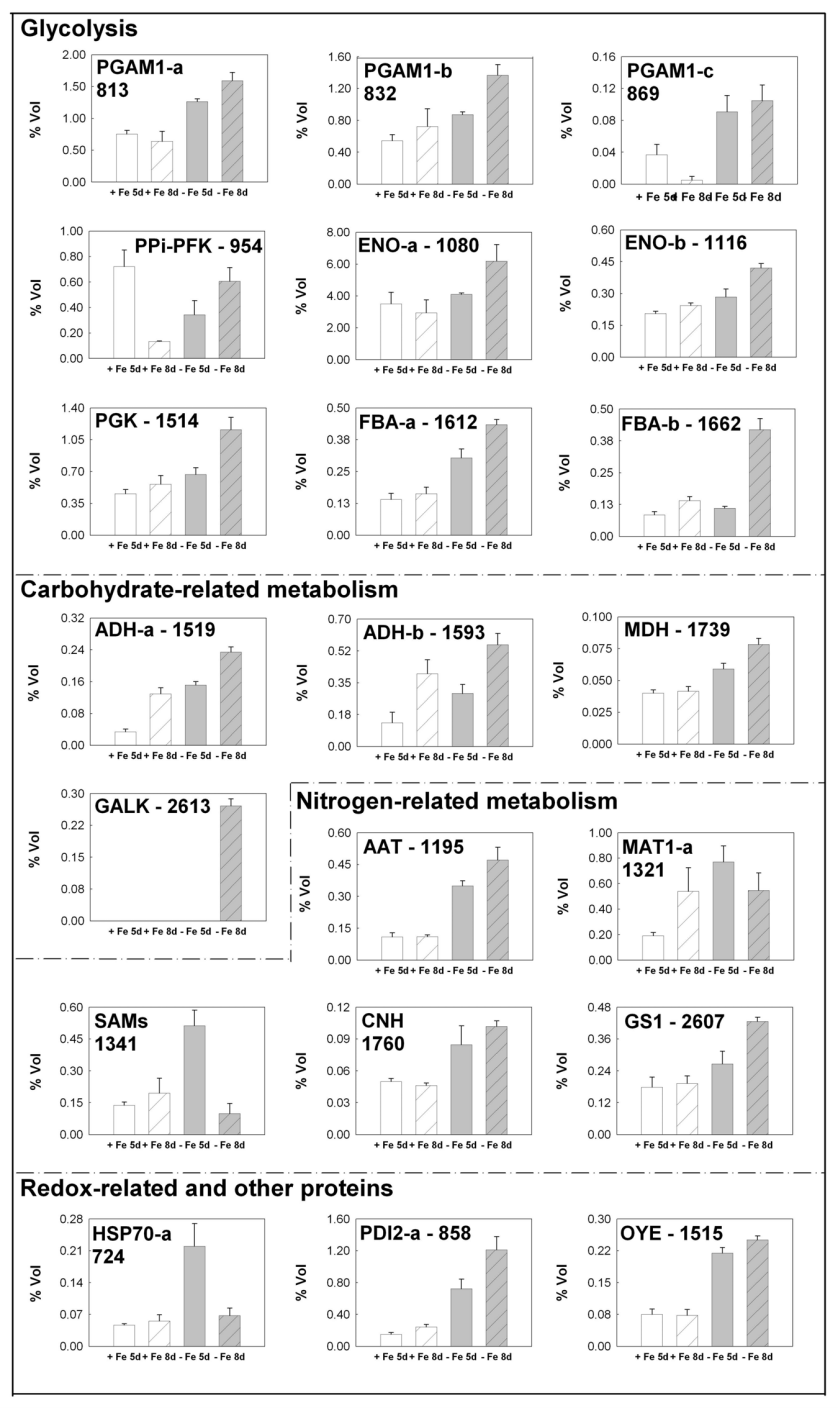
**Changes in the level of the identified proteins whose concentration is increased under Fe deficiency**. Changes in the relative spot volumes of the identified proteins whose concentration is increased in cucumber roots under Fe deficiency. The data were obtained from plants grown for 5, and 8 d in the presence (+Fe) or absence (-Fe) of Fe. Values are the mean ± SE of six 2-DE gels derived from three independent biological samples analyzed in duplicate (n = 6). Numbers identify the spots as reported in Tables 1 and 2.

**Figure 7:**
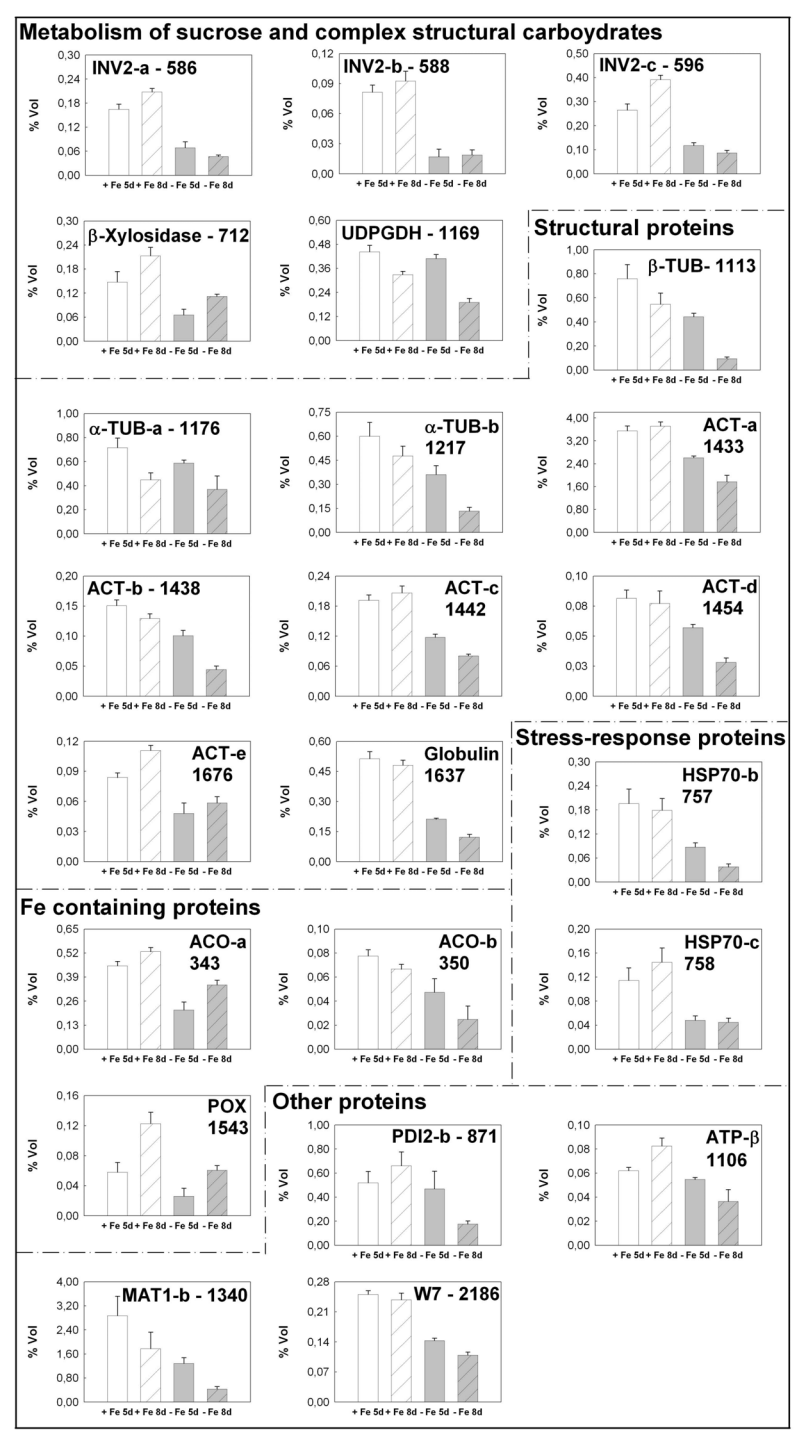
**Changes in the level of identified proteins whose concentration is decreased under Fe deficiency**. Changes in the relative spot volumes of the identified proteins whose concentration is decreased in cucumber roots under Fe deficiency. The data were obtained from plants grown for 5, and 8 d in the presence (+Fe) or absence (-Fe) of Fe. Values are the mean ± SE of six 2-DE gels derived from three independent biological samples analyzed in duplicate (n = 6). Numbers identify the spots as reported in Tables 1 and 2.

Figure 6 reports the changes in the relative spot volumes of proteins that were increased in quantity under Fe deficiency. For most of the proteins there was an increasing trend between the 5^th ^and the 8^th ^day after Fe starvation, indicating that the response lasts for several days after its induction. As stated before, most of these proteins belong to the glycolytic pathway, confirming previous biochemical results showing an increased activity of some of these enzymes. Three proteins decreases to the level of the control only after 8 d of Fe starvation (spots number 724, 1321 and 1341). The first is a heat shock protein with a MW of 70 Kd (HSP70) and its early increase is not easily understood, since other proteins (spots number 757 and 758) identified as HSP70 decrease under Fe starvation (see Table 2 and Figure 7). The other two proteins (spot numbers 1321 and 1341) were identified as S-adenosylmethionine synthase. This enzyme is the starting point of the metabolic pathway for the biosynthesis of nicotianamine [38] and phytosiderophores of the mugineic acid family. Nicotianamine is considered a Fe transporter in Strategy I plants. From the phenotype of the Na-auxotroph tomato mutant *chloronerva *a key role for nicotianamine in the transport of Fe taken up by the roots to the shoots was postulated [39].

Figure 7 reports the changes in the relative spots volume of proteins that were reduced in quantity during Fe deficiency. As stated before, most of these proteins belong to structural proteins or to stress response protein groups. Interestingly, other decreases correspond to enzymes related to carbohydrate metabolism and linked to the biosynthesis of cell wall polysaccharides (spot numbers 586, 588, 596, 712 and 1169) in good agreement with the hypothesis of a recycling of these carbohydrate units. Also, enzymes containing Fe (aconitase, spot numbers 349 and 350 and peroxidase, spot number 1543) are decreased accordingly with a decreased level of Fe in the cell.

## Discussion

In this work we have analyzed the soluble proteins extracted from cucumber roots grown in the presence or in the absence of Fe at two different dates, 5 d and 8 d, by 2-DE. Recently, some proteomic studies on Fe deficiency responses have appeared in the literature [29-31]. The first two papers reported the differential expression of proteins in two tomato lines: the T3238-FER genotype and its Fe uptake-inefficient mutant T3238-fer. The former [29] was a study addressed to the identification of a diverse set of differentially accumulated proteins under the control of FER and/or Fe supply, while the latter [30] was a study on total root proteins extracted from these two tomato genotypes, with the increase/decrease being evaluated in a single date after one week of treatment. The third paper [31] reports changes in the proteomic profiles of sugar beet root tips in response to Fe deficiency and resupply.

In order to correlate the metabolic evidences so far obtained in roots of Fe-deficient plants, we have restricted our research to the soluble cytosolic proteins in order to avoid any interference by other cellular systems. Furthermore, we have applied another restriction by characterizing only those spots which showed a two-fold increase or decrease. Under these experimental conditions, 44 proteins that change their level of accumulation were identified. Twenty-one out of 44 increased their concentration under Fe deficiency. Among these, the majority (42% of the total) are enzymes belonging to the glycolytic pathway, confirming previous biochemical data suggesting the involvement of metabolism, and in particular of glycolysis, in response to Fe deficiency. In fact, previous biochemical evidences had shown that under these growing conditions the activities of hexokinase (HK), ATP-dependent phosphofructokinase-1 (ATP-PFK1), glyceraldehyde 3-phosphate dehydrogenase (GAP-DH) and pyruvate kinase (PK) were increased [18,19,34]. Surprisingly, none of these enzyme was detected in this proteomic study, but other enzymes of this pathway such as PP-dependent phosphofructokinase (PP-PFK), aldolase, phosphoglycerate kinase (PGK), phosphoglycerate mutase (PGM) and enolase were detected and found to be enhanced by Fe deficiency. This discrepancy could be explained by several factors. First of all, it is always risky to strictly link protein levels to their activities: these glycolytic enzymes, in fact, are known to be highly regulated by allosteric mechanisms [23]. In our case, it is thus possible that such mechanisms act in concert with slight increases in the amount of proteins, which might be not considered after the statistical analysis for the subsequent MS analysis. The incomplete match between the levels of some glycolytic enzymes and their activities is also supported by gene expression and the microarray analysis conducted on *Arabidopsis*, that revealed that only ATP-PFK1, PGK, PGM and enolase transcripts increase in Fe-deficient roots after seven days of Fe starvation, while for HK, GAP-DH and PK a decrease was shown, corroborating in some way our proteomic data [28]. Finally, the peculiarities of the electrophoretic approach must be taken into account. For instance, it is possible that some glycolytic enzymes were not considered in this analysis because of the *pI *or the molecular weight ranges employed, comigration phenomena and problems of saturation staining.

The same major discrepancy occurs for the PEPC activity whose increase was around 4 fold in cucumber roots, but it was not detected in this proteomic study. The same discrepancy was also found in the proteomic study carried out on sugar beet root tips [31]. However, the amount of protein as determined by immunochemical identification indicated a consistent increase after 10 d of Fe starvation, while if we compare the times used in this work the enhancement between the control and -Fe conditions was less evident [21] and perhaps below the two fold-increase considered for the successive identification by mass spectrometry. Furthermore, the increase in the activity of PEPC could be related to the complex regulation of this enzyme exerted by the positive allosteric effector Glucose-6-P, whose level has been shown to increase under Fe deficiency [19], and by post-translational regulation [40]. These data are in agreement with the microarray analysis [28] done in *Arabidopsis*, which shows that the PEPC transcript increase occurs only at the 5th d of Fe deficiency, while at the 7th d the transcript is undetectable. While our data on the glycolytic enzymes are in good agreement with those obtained by Rellán-Álvarez et al [31], they agree only in part with those of Li et al [30], since they found that only enolase and triose-P-isomerase increase their level, while, on the contrary, the aldolase activity decrease; from this point of view our data on the involvement of glycolytic enzymes give a much more complete picture. The increase in the glycolytic pathway under Fe deficiency has been confirmed by many biochemical data obtained by several groups [18,19,22] and by the proteomic data described in this work, and is in agreement with the major request of energy, reducing equivalents and carbon skeletons to sustain the greater energetic effort and the request of substrate for the synthesis of the large amount of mRNAs and proteins related to this response [41,42]. Another interesting result is the increase of alcohol dehydrogenase (spot numbers 1519 and 1593) that would confirm the involvement of anaerobic metabolism in response to Fe deficiency [22]. This increase is also in agreement with the microarray study in *Arabidopsis *[28] in which the transcript for the alcohol dehydrogenase was found to be increased.

The metabolic changes induced by Fe deficiency on the protein pattern is not confined only to glycolysis but other pathways seem to be rearranged as a consequence of this stress, as it occurs for instance in the mitochondria [27,33]. In fact, we found that enzymes related to carbohydrate metabolism might be suppressed or increased. In particular, enzymes related to the biosynthesis of cell wall polysaccharides such as invertase, 1,4-β-xylosidase and UDP-Glucose dehydrogenase (UDP-Glc-DH) are decreased (Table 2). The decrease in the biosynthesis of the cell wall polysaccharides in Fe-deficient roots would mean a decrease in carbon flux towards the synthesis of cell wall (more likely less important in these conditions) favoring instead glycolysis and other biosynthetic pathways. Moreover, the cell wall can be considered, in conditions where the photosynthetic apparatus might be damaged or not properly working, as a temporary source of carbohydrates. In order to sustain this change in metabolism we found an increased concentration of galactokinase after 8 d of Fe deficiency, which would channel carbon skeletons originating from cell wall degradation to fuel glycolysis. This enzyme is involved in the metabolism of D-galactose-containing oligo- and polysaccharides and occurs in various plants. The raffinose family of oligosaccharides (RFOs) ranks next to sucrose in their abundance in plant kingdom [43]. Plant cell wall contains numerous polysaccharides which consist of a wide range of different sugar residues. An analysis of *Arabidopsis *identified glucose, rhamnose, galactose, xilose, arabinose and galacturonic and glucuronic acids as the major sugar constituent in the cell wall [44], while a study on the changes of metabolites occurring in sugar beet root tips under Fe deficiency showed a large increase in the RFO sugars [31]. Galactokinase belongs to a sugar-1-P kinase family which account for hydrolysis and recycle of pectic polymers. RFOs might therefore be an important source of rapidly metabolisable carbon other than function as antioxidant [31], (ROS detoxification has been observed in Fe-deficient roots [45]), then, the increase in RFO could help to alleviate ROS damage produced under Fe deficiency. The simultaneous decrease in enzymes involved in the cell wall synthesis might bring to the observed stunting growth of roots under Fe deficiency. Changes in cell wall metabolism has been also observed in Fe-deficient tomato roots [30] and the decrease in invertase activity could, as suggested by Li et al. [30] decrease the relative level of fructose and explain why a down regulation of fructose metabolism was found in these roots.

Another important group of proteins which increase under Fe deficiency is related to nitrogen metabolism (24%). S-adenosylmethionine synthase, alanine aminotransferase, glutamine synthase 1 (the root isoform of GS) and a C-N hydrolase family protein belong to this group. Concerning this group only the S-adenosylmethionine synthase shows a temporal increase, which is limited to the first date of Fe deficiency (Figure 6). This enzyme is involved not only in the biosynthesis of nicotianamine and phytosiderophores of the mugineic acid family [38], but also in the biosynthesis of ethylene, which has been reported to influence the response of Strategy I plants to Fe deficiency [7]. The other three proteins increase at both dates considered. Among them, the most interesting is the C-N hydrolase family protein. In fact, this family of protein includes several enzymes that are involved in nitrogen metabolism and that cleave nitriles as well as amides. Utilization of these nitrogen compounds usually involves several reduction steps. The final step is the assimilation of NH_4_^+ ^or its transfer to various intermediates such as keto acids [46]. It is well known that Fe deficiency leads to an increase in the organic acid level which play different roles one of which is linked to the synthesis of amino acids [25]. Our study also shows a decrease in the cytoskeleton proteins actin and tubulin along with the storage protein globulin (Table 2 and Figure 7). An intriguing hypothesis we can drive from these results is that all these proteins might be recycled under Fe deficiency and used as a source of amino acids, carbon skeletons and nitrogen. This could be in agreement with the increase in the C-N hydrolase protein family and, even if with contrasting results, with changes in two spots identified as protein PDIs. PDIs catalyses the rearrangement of disulfide bridges of proteins [47] and in *Arabidopsis *these family of proteins is encoded by 12 genes [48]. While spot number 858 (Table 1 and Figure 6) increases, the other one, spot number 871 (Table 2 and Figure 7) decreases, especially after 8 d. Contrasting results have been found also for spots identified as heat shock proteins, where in one case (spot number 724) we found an increase while in two cases (spot numbers 757 and 758), on the contrary, a decrease was observed. PDIs and HSP70 are involved in the mechanism(s) of protein folding as molecular chaperones (HSP70) and protein folding catalysts (PDIs) so assuring a proper fold of nascent polypeptides into functional proteins. This variability could be associated with a change in the ratio between biosynthesis an degradation of proteins that could bring to a release of amino acids that might serve both as nitrogen and carbon sources. We are aware that the hypothesis is speculative, but the data obtained in this proteomic study support it. Furthermore, other data obtained in our laboratory (manuscript in preparation) show a decrease in the activity of enzymes of the nitrogen assimilatory pathway, since some of them, such as nitrate reductase and nitrite reductase, are Fe-dependent.

## Conclusions

In conclusion, the data obtained in this proteomic profiling study confirm some metabolic changes occurring as a response to Fe deficiency. In particular, our data support the increase in the glycolytic flux and in the anaerobic metabolism to sustain the energetic effort Fe-deficient plants must undertake. In fact, Fe deficiency leads to an impairment of the mitochondrial respiratory chain, so the cell must overcome this problem by activating alternative pathways to sustain the energetic requirement and the NAD(P)H turnover [33,49]. We also found a decrease in the amount of enzymes linked to the biosynthesis of complex carbohydrates of the cell wall, and, on the other hand, an increase in enzymes linked to the turnover of proteins. In a scenario in which the production of new carbon skeletons is strongly impaired by a less efficient photosynthetic apparatus, the plant must face the increased demand of energy and organic compounds. This "cellular effort" seems to be comparable with that occurring in the mammalian muscles in which a strong energetic effort, caused by an enhanced muscular activity, stimulate the anaerobic pathway to produce energy [27]. In Fe-deficient plants, the effort is much more complex, since the contribution of photosynthesis is poor and the plant must recover carbon skeletons from other sources to sustain metabolism. We are aware that more work is necessary to better understand what is going on under Fe deficiency, but the data obtained in the present proteomic work along with those on metabolic activities could cast new light on the responses induced by Fe-deficient plants.

## Methods

### Plant material and growth conditions

Cucumber (*Cucumis sativus *L. cv. Marketmore '76 from F.lli Ingegnoli, Milan) seeds were sown in agriperlite, watered with 0.1 mM CaSO_4_, allowed to germinate in the dark at 26 °C for 4 d. Thirty seedlings were transferred to a 10 L tank for hydroponic culture. The nutrient solution had the following composition: 2 mM Ca(NO)_3_, 0.75 mM K_2_SO_4_, 0.65 mM MgSO_4_, 0.5 mM KH_2_PO_4_, 10 μM H_3_BO_3_, 1 μM MnSO_4_, 0.5 μM CuSO_4_, 0.5 μM ZnSO_4_, 0.05 μM (NH_4_)Mo_7_O_24 _and 0.1 mM Fe-EDTA (when added). The pH was adjusted to 6.2 with NaOH. Aerated hydroponic cultures were maintained in a growth chamber with a day/night regime of 16/8 h and a photosynthetic photon flux density (PPFD) of 200 μmol m^-2 ^s^-1 ^at the plant level. The temperature was 18 °C in the dark and 24 °C in the light. The effect of different treatments at the root level was determined after 5 and 8 d. A scheme of the growing condition is reported in Figure 1A.

### Semiquantitative RT-PCR

Root tissues were ground in liquid nitrogen using mortar and pestle, and total RNA was extracted using Trizol^® ^reagent (Invitrogen, Milano, Italy). First-strand cDNA synthesis was carried out using the iScript™cDNA Synthesis Kit (Bio-Rad, Milano, Italy) according to the manufacturer's instructions. Actin was used as house keeping gene. Semiquantitative RT-PCR was carried out on the first-strand cDNA and the identity of the amplified fragments verified by sequencing both strands. To detect differences in the cDNA expression level for each sample set, a variable number of amplification cycles, between 20 and 24 depending on gene templates, were tested. The thermal cycle program was: one initial cycle at 94°C for 5 min, followed by cycles at 94°C for 30 sec, 56°-60°C for 1 min, 72°C for 1 min, with 20-24 cycles for TDFs selected for the RT-PCR analysis, all followed by a final 72°C elongation cycle for 5 min. The amplified products were run on a 1% agarose gel without ethidium bromide. The gels were incubated in Tris-HCl 1 mM pH 8, EDTA 0,1 mM adding 1‰ of Vistra Green Nucleic Acid Stain (GE Healthcare Life Sciences, USA), as fluorescent stains, for 30 min. Then, gels were scanned and bands were detected with the Typhoon 9200 high performance laser scanning system (GE Healthcare Life Sciences, USA).

For the internal reference amplification profile, the constitutive expression level was compared for each reaction by using primers against the actin transcript of cucumber (*Csactin*, Genbank accession no AB010922) according to Waters et al [7]. RT-PCR analysis was also performed for *CsFRO1*, *CsIRT1 *and *CsHA1 *(Genbank accession nos. AY590765, AY590764 and AJ703810, respectively) using specific primers according to Santi et al. [50] and Waters et al., [7]. The validation of all the steps of the experiment was done with three independent biological replicates each of them with two technical replicates.

### Extraction of protein samples for 2-DE analysis

Roots of plants grown in the presence or absence of Fe were harvested, rinsed in distilled H_2_O and homogenized in a buffer containing 50 mM TRIS-HCl (pH 7.5), 10 mM MgCl_2_, 10% (v/v) glycerol, 1 mM EDTA. 14 mM β-mercaptoethanol, 1 mM phenylmethylsulphonyl fluoride (PMSF) and 10 μg ml^-1 ^leupeptin were added to avoid or minimize proteolysis [according to 51]. A ratio of 3 ml of buffer per 1 g of roots was used. The homogenate was centrifuged at 13 000 *g *for 15 min and the supernatant was again centrifuged at 100 000 *g *for 30 min. Proteins were then precipitated by adding four volumes of pre-cooled 12.5% TCA in acetone and incubating them at -20°C overnight. Precipitated proteins were recovered by centrifuging at 13 000 *g *at 4 °C for 30 min and then washed two times with cold 80% (v/v) acetone. The final pellet was dried under vacuum and dissolved in IEF buffer [7 M urea, 2 M thiourea, 3% (w/v) CHAPS, 1% (v/v) NP-40, 50 mg mL^-1 ^DTT and 2% (v/v) IPG Buffer pH 4-7 (GE Healthcare Life Sciences, USA)] by vortexing and incubating for 1 h at room temperature. Samples were centrifuged at 10 000 *g *for 10 min and the supernatants stored at -80°C until further use. The protein concentration was determined by 2-D Quant Kit (GE Healthcare Life Sciences, USA). For each condition, three biological replicates were obtained.

### 2-DE analysis

Protein samples (400 μg) were loaded on pH 4-7, 24 cm IPG strips passively rehydrated overnight in 7 M urea, 2 M thiourea, 3% (w/v) CHAPS, 1% (v/v) NP-40, 10 mg mL^-1 ^DTT and 0.5% (v/v) IPG Buffer pH 4-7. IEF was performed at 20 °C with current limit of 50 μA/strip for about 50 kVh in an Ettan IPGphor (GE Healthcare Life Sciences, USA). After IEF, strips were equilibrated by gentle stirring for 15 min in equilibration buffer [100 mM Tris-HCl pH 6.8, 7 M urea, 2 M thiourea, 30% (w/v) glycerol, 2% (w/v) SDS] supplemented with 0.5% (w/v) DTT for disulfide bridge reduction and for an additional 15 min in the same equilibration buffer supplemented with 0.002% (w/v) bromophenol blue and 4.5% (w/v) iodoacetamide for cysteine alkylation. Second-dimensional SDS-PAGE was run in 10% acrylamide gels using the ETTAN DALT*six *apparatus (GE Healthcare Life Sciences, USA). Running was first conducted at 5 W/gel for 30 min followed by 15 W/gel until the bromophenol blue line ran off. For each biological replicates two technical replications were performed (n = 6).

### Protein visualization and data analysis

Gels were stained using the colloidal Coomassie Brilliant Blue G-250 (cCBB) procedure, as previously described by Neuhoff et al. [52]. The gels were scanned in an Epson Expression 1680 Pro Scanner and analyzed with ImageMaster 2-D Platinum Software v6.0 (GE Healthcare Life Sciences, USA). Automatic matching was complemented by manual matching. Molecular weights of the spots were estimated using a migration wide range standard (MW 6.500 - 205.000, GE Healthcare), while *pI *was determined according to the strip manufacturer's instructions (GE Healthcare Life Sciences, USA).

During this analysis only spots showing at least a two-fold change in expression and having a relative spot volume average (% Vol) larger than 0.08 in at least one of the four treatments were considered for successive steps. In order to find differentially expressed proteins, all values were log(z+1) transformed and a Two-way ANOVA (*p *<0.001), with Fe and days of treatment as factors, was carried out. Significant differences linked to the factor Fe were analyzed through a two-way hierarchical clustering methodology, using the software PermutMatrix as previously described by Negri et al [53].

### Protein in-gel digestion and LC-ESI-MS/MS analysis

Spots excised from the cCBB gels were digested as described by Prinsi et al [54]. The LC-ESI-MS/MS experiments were conducted using a Surveyor (MS pump Plus) HPLC system directly connected to the ESI source of a Finnigan LCQ DECA XP MAX ion trap mass spectrometer (ThermoFisher Scientific Inc., Waltham, USA). Chromatography separations were obtained on a reverse phase C18 column (200 μm I.D × 150 mm length, 5 μm particle size), using a gradient from 5% to 80% solvent B [solvent A: 0.1% (v/v) formic acid; solvent B: ACN containing 0.1% (v/v) formic acid] with a flow of 2.0 μl/min. ESI was performed in positive ionization mode with spray voltage and capillary temperature set at 2.5 kV and at 220 °C, respectively. Data were collected in full-scan and data dependent MS/MS mode with a collision energy of 35% and a dynamic exclusion window of 3 min.

Spectra were searched by TurboSEQUEST^® ^incorporated in BioworksBrowser 3.2 software (ThermoFisher Scientific Inc., Waltham, USA) against the *Cucumis *protein subset, *Cucumis sativus *EST subset and against the protein NCBI-nr database, all downloaded from the National Center for Biotechnology Information http://www.ncbi.nlm.nih.gov/. The searches were carried out assuming parent ion and fragment ion mass tolerance of ± 2 Da and ± 1 Da, respectively, two possible missed cleavages per peptide, fixed carboxyamidomethylation of cysteine and variable methionine oxidation. Positive hits were filtered on the basis of peptide scores [Xcorr ≥ 1.5 (+1 charge state), ≥ 2.0 (+2 charge state), ≥ 2.5 (≥ 3 charge state), peptide probability < 1 × 10^-3^, ΔCn ≥ 0.1 and Sf ≥ 0.70]. If needed, identified peptides were subjected to a protein similarity search performed by alignment analyses against the NCBI-nr database using the FASTS algorithm http://fasta.bioch.virginia.edu/fasta_www2/[55]. Theoretical molecular masses and p*Is *of characterized proteins were calculated by processing sequence entries at http://www.expasy.org/tools/pi_tool.html.

## Authors' contributions

SD carried out protein extraction, 2-DE gel analysis, statistical analysis and drafted the manuscript. BP carried out protein characterization by LC-ESI-MS/MS, analysed the MS data. ASN carried out the clustering and statistical analysis. GV carried out the RT-PCR analysis. LE coordinated the 2-DE gel analysis and the LC-ESI-MS/MS analysis. GZ participated in the strategic planning of the work, data analysis and writing the manuscript. All the authors contributed to the discussion of the results and took part to the critical revision of the manuscript. All authors read and approved the final manuscript.

## Supplementary Material

Additional file 1 **List of the identified proteins by LC-ESI-MS/MS and bioinformatics analyses**. The table shows the sequence of all the peptides identified by MS/MS fragmentation and the associated statistical information obtained from database searches conducted by BioworksBrowser using TurboSEQUEST^® ^software. For each identified protein, statistical information related to direct protein database search or to alignment analysis of the identified peptides by FASTS software are reported. **Spot ID**: spot identifier number. **Protein A.N**.: protein NCBI accession number (version). **DB**: database downloaded from NCBI: NR = protein non-redundant database; NRc: subset of *Cucumis *genus proteins; EST: subset of *Cucumis sativus *ESTs. **n. pep**.: number of the unique peptides used to identify the protein. **a.a. cov. (%)**: sequence coverage %. **Sf (pro)**: protein SEQUEST Sf score. **FASTS (*E*) value**: FASTS expectation (*E*) value of the entry resulting from the alignment of the peptides against NCBI non-redundant database. **Hom. Protein A.N**.: homologous protein NCBI accession number (version). **EST A. N**.: EST NCBI accession number (version). **Peptide**: sequence of the identified peptide; the symbol M* indicates oxidized methionine. **MH+**: molecular mass of the peptide; **z**: charge state of the peptide. **Sf (pep)**: SEQUEST Sf score of the peptide. **Xcorr**: SEQUEST cross-correlation value. **ΔCn**: delta correlation value **Sp**: SEQUEST preliminary score. **(a)**: partial sequence. **(b)**: mature form. Click here for file
